# Synovial lymphoid neogenesis is associated with IL-23 expression and disease activity in rheumatoid arthritis

**DOI:** 10.1186/1479-5876-9-S2-P33

**Published:** 2011-11-23

**Authors:** Juan D Cañete, Raquel Celis, Julio Ramírez, Sara Marsal, Gabriela Ávila, Raimón Sanmartí, José L Pablos

**Affiliations:** 1 Arthritis Unit, Rheumatology Dept., Hospital Clinic of Barcelona and IDIBAPS, Barcelona, Spain; 2 Rheumatology Unit, Hospital de la Vall d’Hebró, Barcelona, Spain; 3 Instituto de Investigación Hospital 12 de Octubre (I+12), Madrid, Spain

## Objective

We analyzed whether LN is associated with specific patterns of inflammatory cytokine expression in synovial tissue (ST), and the potential association of cytokine expression with disease activity and response to therapy.

## Methods

ST samples were obtained by arthroscopy from the inflamed knee of 62 RA patients. A second biopsy was obtained after a mean of 8±5 months of treatment in 21 patients who started TNF blockers. ST samples were immunostained with CD3 (T cell), CD20 (B cell), and MECA-79 (high endothelial vessels). Total ST mRNA was extracted and gene expression of CCR7, LT-beta, IL-7, IL-10, IL-17A, IL-21, IL-22, IL-23, TNF-alpha, IL-1b, and IL-6 was measured by quantitative real-time PCR. Clinical and biological data were collected at inclusion and after a median follow-up of 2.3 years.

## Results

28 out of 62 patients (45.2%) had LN, which was associated with a significantly-higher expression of three key molecular markers of LN: CCR7, IL-7 and LT-beta (p<0.037, p<0.038, and p=0.009, respectively). LN-positive patients also had higher IL-23 (p=0.035) and higher DAS28 score, both at inclusion and at the end of follow-up (p=0.055 and p=0.037, respectively). In the group of patients with ST before and after TNF blocker therapy, EULAR good response was associated with IL-10 reduction (p=0.035). In the entire group, EULAR response correlated with IL-1b expression (P=0.014).

## Conclusion

RA patients with LN were characterized by higher expression of key molecular markers of LN, IL-23 and higher disease activity. A significant reduction in IL-10 expression after therapy with TNF-blockers was associated with good EULAR response.

